# Seed Germination Ecology of *Sonchus asper* and *Sonchus oleraceus* in Queensland Australia

**DOI:** 10.3390/plants13233451

**Published:** 2024-12-09

**Authors:** Yun Lee, Gulshan Mahajan, Rita Beregszaszi, Bhagirath Singh Chauhan

**Affiliations:** 1 University of Queensland, Gatton, QLD 4343, Australia; y.lee4@uq.net.au (Y.L.); ritaberegszaszi29@gmail.com (R.B.); 2 Centre for Crop Science, Queensland Alliance for Agriculture and Food Innovation (QAAFI), University of Queensland, Gatton, QLD 4343, Australia; 3 Centre for Crop Science, Queensland Alliance for Agriculture and Food Innovation (QAAFI), School of Agriculture and Food Sustainability (AGFS), University of Queensland, Gatton, QLD 4343, Australia; b.chauhan@uq.edu.au

**Keywords:** burial depth, salt stress, temperature, water stress, weed ecology

## Abstract

*Sonchus asper* and *S. oleraceus* are among the most problematic broadleaf weeds in eastern cropping systems of Australia. This study investigated the seed germination ecology of *S. asper* and *S. oleraceus*. The study hypothesized that *S. asper* may have greater ecological advantages under adverse environmental conditions compared to *S. oleraceus*. Results showed that *S. asper* consistently outperformed *S. oleraceus* across different light regimes and stress conditions. At a lower temperature regime of 15/5 °C, seed germination of *S. oleraceus* decreased by 19% compared to *S. asper*. Germination of *S. oleraceus* significantly declined under dark conditions, while over 90% of *S. asper* seeds germinated under both light/dark and dark conditions. Under water stress (osmotic potential of −0.4 MPa), *S. oleraceus* germination dropped by 74% compared to *S. asper*, indicating *S. asper*’s superior drought tolerance. Both species exhibited moderate salinity tolerance (40 mM NaCl) to germinate, highlighting their potential to invade saline cropping environments. The burial study revealed that *S. oleraceus* had higher germination at the soil surface, but no germination occurred from 4 cm, while 23% of *S. asper* seeds still emerged from that depth. The burial depth required to inhibit 50% emergence of *S. asper* and *S. oleraceus* was 3.3 cm and 0.3 cm, respectively. These findings highlight *S. asper*’s greater adaptability to low temperatures, burial depth, and stress conditions than *S. oleraceus*. The insights from this study can inform agronomic strategies, including tillage depth and mulching, to mitigate the impact of these invasive species on Australian cropping systems.

## 1. Introduction

*Sonchus asper* L. (spiny sowthistle) and *Sonchus oleraceus* L. (annual sowthistle) are two closely related species that have emerged as significant threats to cropping regions in eastern Australia [1]. *Sonchus oleraceus* has softer, less spiny leaves compared to *S. asper***,** which has thick, spiny leaves with a more rigid structure [2]. Both species are highly competitive, reducing crop yields by aggressively outcompeting crops for resources such as water, nutrients, light, and space [1]. The rapid growth of *S. oleraceus* allows it to dominate fields quickly, particularly in winter cropping systems [3] (Steinmaus et al. 2000). *Sonchus asper*’s greater tolerance to environmental stresses enables it to persist in areas where other weeds might fail, maintaining competitive pressure even under harsh conditions [4].

In Australian grain regions, infestations of *Sonchus* species can lead to wheat yield losses of up to 50%, depending on their density and emergence timing [5]. The persistence of *Sonchus* spp. in fallows can also interfere with subsequent crop planting, leading to increased management costs and reduced farm profitability [6]. These yield losses are further exacerbated by the development of herbicide resistance, particularly to glyphosate, which makes these species increasingly difficult to control [7,8,9]. As a result, controlling *Sonchus* species has become more challenging in eastern Australia, necessitating the development of ecological weed control solutions to mitigate their impact.

Understanding the germination ecology of *S. asper* and *S. oleraceus* in eastern Australia is crucial for diving deeper into their biology and behavior under environmental stresses, which can aid in developing integrated management strategies [10]. Given their adaptability, competitive nature, and growing significance as invasive weeds, comparative seed germination ecology studies can provide valuable insights for more effective and sustainable weed management. Such knowledge may help reduce yield losses due to infestation, preserve biodiversity, and ensure the long-term sustainability of eastern Australia’s landscapes [11,12].

Understanding the germination behavior of both species in relation to temperature and light is essential for managing these weeds. Both species are sensitive to environmental conditions, which affect their germination, growth, and persistence [13]. Variations in temperature may influence the germination rate and growth cycles of both weeds [14,15]. *Sonchus oleraceus* is capable of germinating over a broader temperature range (10–35 °C), allowing it to thrive across various climatic conditions of eastern Australia [15]. However, information on the ideal temperature for *S. asper* germination in the eastern cropping region is limited. Knowledge of the temperature preferences of both species can guide the timing of weed control measures in winter crops and fallows.

Previous studies have reported that *S. oleraceus* can germinate under a range of light conditions, demonstrating its adaptability to disturbed environments with inconsistent light availability [14]. However, there is limited information on the light and dark requirements for *S. asper* germination in this region. This knowledge could inform management practices such as mulching or adjusting planting densities to suppress weed growth. In the context of climate change, understanding how these species respond to changes in temperature and light will be critical for predicting weed emergence and optimizing control strategies [16].

*Sonchus oleraceus*, often found in wetter environments, may experience reduced germination and growth under low osmotic potential conditions, while *S. asper*, which tolerates harsher environments, may exhibit greater resilience to drought and salinity [4]. Understanding the impact of osmotic stress on both species can help predict how they will behave in diverse climates, from humid coastal areas to arid inland regions. This knowledge is vital for developing targeted weed management strategies, especially in fields affected by water scarcity or saline conditions.

Studying the germination behavior of *S. asper* and *S. oleraceus* under salt stress is also important due to the increasing soil salinity impacting agriculture in eastern Australia [17,18]. This information can guide the development of targeted weed control strategies to prevent the spread of these species in saline soils, contributing to the creation of effective, proactive management practices. Further, understanding how both species respond to varying burial depths is essential for predicting their persistence in soil seed banks and their potential to invade fields. This knowledge is vital for designing effective weed management practices, such as tillage or seedbed preparation, to minimize weed establishment and reduce their impact on crop yield losses [19].

It is hypothesized that the two *Sonchus* species may exhibit different germination behaviors across a range of temperatures, light conditions, salt and water stress conditions, and burial depths. This study aims to understand how both species respond to varying environmental conditions, which is crucial for developing targeted and effective weed management strategies.

## 2. Materials and Methods

### 2.1. Seed Description

A series of experiments was conducted twice in 2023 at the Weed Science Laboratory of the University of Queensland, Australia. Fresh seeds of both *Sonchus* spp. were collected from mature plants grown during the winter of 2022 under field conditions at the Gatton Research Farms of the University of Queensland. After collection, the seeds were cleaned, dried, and stored at room temperature (25 °C) in the laboratory until the experiments commenced. The 100 seed weights of *S. oleraceus* and *S. asper* were 0.024 g and 0.031 g, respectively.

### 2.2. General Germination Test Protocol

For the petri dish experiments, 25 seeds were placed in 9 cm Petri dishes lined with a double layer of filter paper (Whatman Grade 1, Global Life Sciences Solutions Operations UK Ltd., Sheffield, UK). The petri dishes were sealed in plastic bags and incubated under different treatments, each programmed with a 12 h dark/light cycle. Fluorescent lamps, emitting 85 µmol m⁻^2^ s⁻^1^ of light, illuminated the incubators. The experiments ran for three weeks, with each one repeated after the first trial was completed. In our preliminary trials on *Sonchus*, which were conducted for five weeks, there was no further germination after 3 weeks. Therefore, in this study, we decided on a three-week duration.

### 2.3. Effect of Temperature and Light

For the light/dark experiments, seeds of both *Sonchus* species were incubated under a range of alternating day/night temperatures: 15/5 °C, 25/10 °C, 25/15 °C, 30/20 °C, and 35/25 °C, all with a 12 h light/dark cycle. In the dark treatment, petri dishes were wrapped in 3 layers of aluminum foil and placed in the incubators set to the same temperature regimes. These temperature conditions were selected to simulate the environmental variability found in Queensland, ensuring that the data would be applicable to real-farming situations.

### 2.4. Effect of Osmotic Stress

Osmotic stress was assessed using five water potential levels (0, −0.1, −0.2, −0.4, and −0.8 MPa). Each petri dish was moistened with 5 mL of polyethylene glycol (PEG) solution according to treatment, after which the seeds of both species were placed in the petri dishes. The germination study was conducted in an incubator set at 25/15 °C with a 12 h light/dark cycle.

### 2.5. Effect of Salt Stress

To evaluate the salinity stress, sodium chloride (NaCl, Sigma-Aldrich^®^, St. Louis, MO, USA) solutions at five concentrations (0, 20, 40, 80, and 160 mM) were used in place of water (5 mL) in the petri dishes. These concentrations were chosen to reflect the typical salinity levels found in Australia’s major cropping regions. Like the water stress trial, the germination study was conducted in an incubator set at 25/15 °C with a 12 h light/dark cycle.

### 2.6. Effect of Burial Depth on Seedling Emergence

To evaluate seedling emergence, 25 seeds from each species were planted at six distinct burial depths (0, 0.5, 1, 2, 4, and 8 cm) in plastic pots with a 10 cm diameter. The soil used was clay loamy, with 2.7% organic matter and a pH value of 7.2. The soil was sterilized to avoid the background seed bank. Seedling emergence was recorded 35 days after sowing.

### 2.7. Statistical Analyses

All experiments were arranged in a completely randomized design with three replications. Each experiment was repeated after the first run, and the data presented represent the averages of both runs. No significant time-by-treatment interactions were detected, as verified by ANOVA (using OPStat software: https://opstat.somee.com/opstat/, accessed on 28 March 2024). ANOVA was utilized to identify significant treatment and interaction effects at *p* ≤ 0.05. When treatments were significant, means were separated using Fisher’s protected LSD test at *p* ≤ 0.05. Nonlinear regression analysis was used to examine the relationships between germination and salt concentration (a three-parametric sigmoid model) and between emergence and burial depth (a three-parametric logistic model) using SigmaPlot (v 15.0), described by the following equations:

(1) $$y=y_{max}\;/\;[1+exp(-(x-x_{50}\;)\;/\;y_{rate}))]$$

(2) $$y=y_{max}\;/\;{\left((1+x)\;/x_{50}\;\right)}^{y_{rate}}$$

where *y* represents the total germination or emergence percentage at a given salt concentration or burial depth (*x*), *y*_max_ is the maximum germination or emergence (%), *x*_50_ is the level at which 50% inhibition of maximum germination/emergence occurs, and *y*_rate_ denotes the slope of the curve.

These models provided a strong fit (as indicated by their *R*^2^ value) to the data, offering valuable insights into how salt concentration or burial depth affects the germination of *Sonchus* spp. We tried to fit a regression model for osmotic potential, but the equation did not fit. Therefore, we explained the results of the osmotic potential trial using the LSD value.

## 3. Results and Discussion

### 3.1. Effect of Temperature and Light

**Figure 1:**
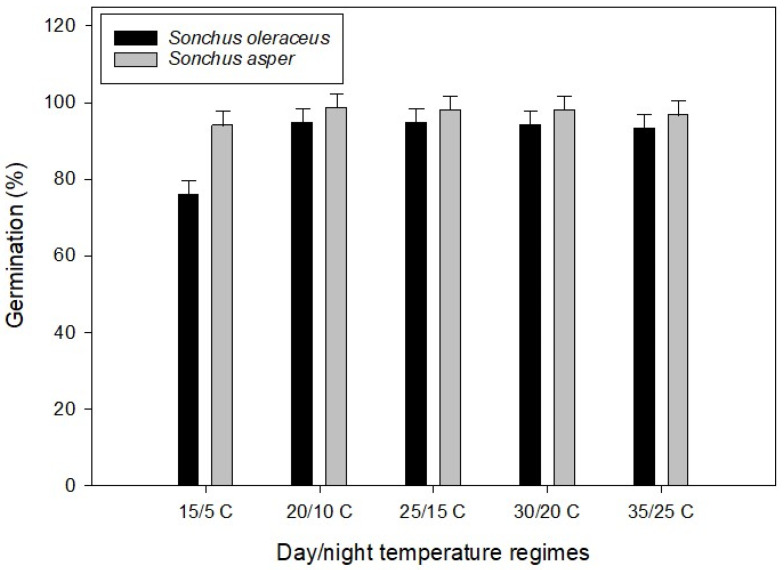
Effect of alternating day/night temperatures (15/5 to 35/25 °C) on the germination of Sonchus oleraceus and *Sonchus asper*. Seeds were incubated for 21 d. Error bars represent LSD values at a 5% level of significance.

A significant interaction (*p* ≤ 0.05) between temperature regimes and *Sonchus* spp. was observed for seed germination (Figure 1). Both species exhibited similar germination patterns across all temperature regimes except at 15/5 °C. At this temperature (15/5 °C), *S. oleraceus* germination decreased by 19% compared to *S. asper*. These results indicate that while *S. asper* is unaffected by lower temperatures, *S. oleraceus* may experience reduced germination under severe winter conditions or in colder regions.

Previous studies have also observed reduced germination of *S. oleraceus* by 62–65% at a temperature regime of 15/5 °C [13,19]. Our study supported these findings, demonstrating reduced germination of *S. oleraceus* at 15/5 °C. Additionally, we found that, unlike *S. oleraceus*, the germination of *S. asper* was not reduced at a low-temperature regime (15/5 °C) compared with high-temperature regimes. As supported by current and earlier research, *S. oleraceus* can germinate across a broad temperature range [15,19]. However, some researchers [20] also noted that over 90% of *S. oleraceus* seeds germinated between 5 °C and 35 °C, which differs from our observations at 15/5 °C, suggesting population-specific and environmental variability. While there is limited research comparing *S. asper* and *S. oleraceus*, our results contribute valuable insights. Like *S. oleraceus*, *S. asper* exhibits similar temperature preferences, though it demonstrates better germination under extreme cold conditions (15/5 °C).

**Figure 2:**
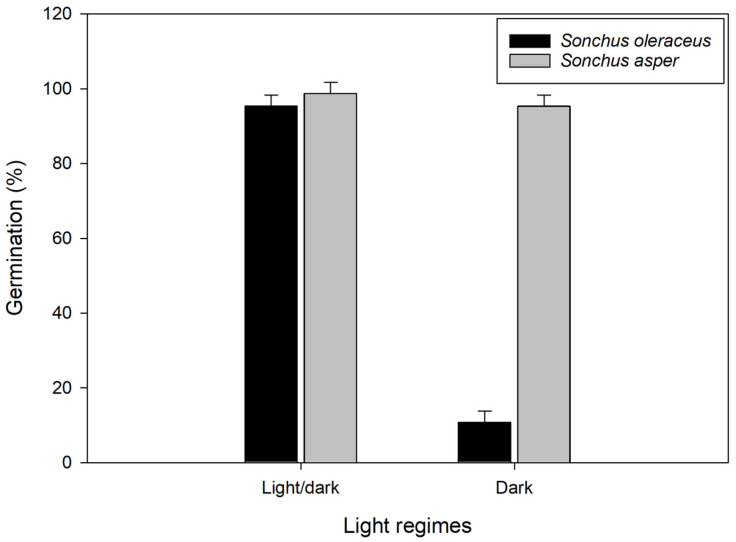
Effect of alternating light/dark treatments (12 h photoperiod) and complete dark (24 h photoperiod) on *Sonchus oleraceus* and *Sonchus asper* germination. Seeds were incubated for 21 d at an alternating temperature of 25/15 °C with a 12 h light/dark cycle. Error bars represent LSD values at a 5% level of significance.

A significant interaction (*p* ≤ 0.05) between light conditions and *Sonchus* spp. was also observed (Figure 2). While light regimes had no effect on *S. asper* germination, *S. oleraceus* germination significantly decreased under dark conditions compared to the light/dark cycle. These results are in agreement with previous studies in which it was reported that *S. oleraceus* had reduced germination under complete darkness compared with a light/dark regime [13,19].

These findings suggest that *S. oleraceus* is less likely to germinate in dark conditions, such as those created by crop residues in no-till systems. However, seeds exposed to light through shallow tillage are more likely to germinate. This insight underscores the importance of considering light exposure when developing weed management strategies. Targeted practices, such as mulching, shading, or adjusting tillage depth, can suppress seed germination of these species by controlling light availability.

Overall, our results suggest that *S. oleraceus* prefers light/dark conditions for optimal germination. Therefore, strategic tillage that buries *S. olearceus* seeds deeper in the soil may help reduce germination rates.

### 3.2. Effect of Osmotic Stress

**Figure 3:**
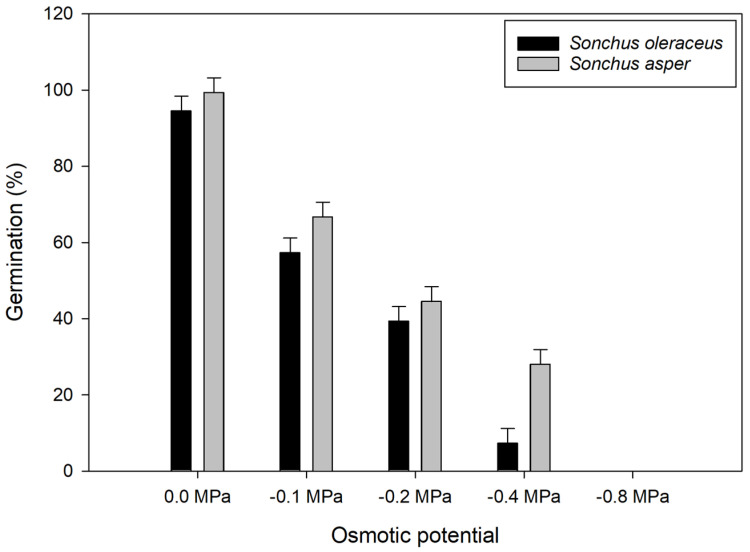
Effect of osmotic potential on *Sonchus oleraceus* and *Sonchus asper* germination at alternating day/night temperatures of 25/15 °C under a 12 h photoperiod. Seeds were incubated for 21 d at an alternating temperature of 25/15 °C with a 12 h light/dark cycle. Error bars represent LSD values at a 5% level of significance.

Seed germination was influenced by the interaction between *Sonchus* species and osmotic potential (Figure 3). As osmotic potential decreased from 0 to −0.8 MPa, germination of both species declined and was absent at −0.8 MPa. Both *Sonchus* spp. behaved similarly for germination at each osmotic potential except at −0.4 MPa, where *S. oleraceus* germination was reduced by 74% compared to *S. asper*.

These observations suggest that *S. asper* is more tolerant to water stress of −0.4 MPa than *S. oleraceus*. These findings have significant implications for the invasion and competition between these two species. With its higher drought tolerance, *S. asper* is likely to be more resilient in water-stressed regions, giving it a competitive edge over *S. oleraceus*. This could lead to increased invasiveness and spread of *S. asper*, particularly in environments where water scarcity limits the establishment of other species. A previous study showed that the germination rate of *S. asper* declined with increasing hydric pressure but retained a higher germination capacity than *S. oleraceus* [4]. In another study, the germination rate of *S. oleraceus* dropped significantly to 11% when osmotic pressure reached −0.6 MPa, illustrating the species’ vulnerability to water stress [19].

### 3.3. Effect of Salt Stress

**Figure 4:**
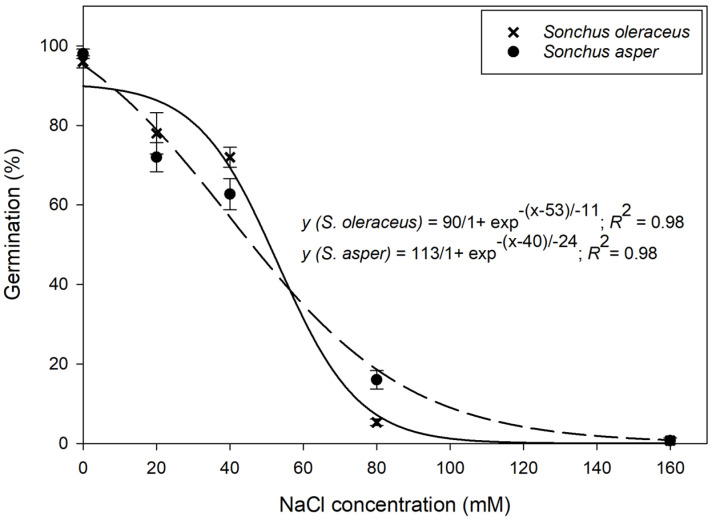
Effect of sodium chloride (NaCl) concentration on the germination of *Sonchus oleraceus* and *Sonchus asper* at alternating day/night temperatures of 25/15 °C under 12 h photoperiod. Error bars represent the standard error of mean (*n* = 6). Data were subjected to a three-parametric sigmoid curve.

Seed germination of *Sonchus* species showed a sigmoid response to varying salt concentrations (Figure 4). The NaCl concentration required to inhibit 50% germination of *S. oleraceus* and *S. asper* was 53 mM and 40 mM, respectively. These results suggest that both species responded differently to increasing NaCl concentrations. The notable germination of both weeds at 40 mM NaCl suggests that under saline soil conditions in Australia, both species can thrive and compete with crops.

Previous research has highlighted *S. oleraceus*’s salt tolerance [19]. Our findings build on this by comparing both *S. oleraceus* and *S. asper*, revealing that while both species exhibit similar responses to salinity stress, *S. asper* demonstrates a slight advantage in higher salinity environments, with 16% germination at elevated salt concentrations (80 mM NaCl).

This differential tolerance between the two species has important agronomic implications, requiring alternative management tactics for identifying and controlling the species in different landscapes [17]. A previous study found that moderate salinity stress (40–60 mM) did not adversely affect the root growth of *S. oleraceus*, suggesting that root phenotypes play a key role in salinity adaptation [21]. Although there is limited research on *S. asper*, the similar responses of both species to salinity stress suggest they may share similar physiological adaptations. Further investigation into the root structures and cellular mechanisms of these species could provide insight into their ability to thrive in saline environments.

Moreover, the high salinity tolerance of *Sonchus* species is not entirely disadvantageous from an ecological perspective. Some researchers discussed how certain weed species, including *S. oleraceus*, could contribute to environmental rehabilitation in saline soils due to their adaptability [22]. This opens new possibilities for using these species in ecological restoration projects, where their tolerance to harsh conditions could aid in the regeneration of degraded lands. However, the dual role of *Sonchus* species as adaptable weeds and potential ecological rehabilitators calls for careful management. In environments with fluctuating salinity, the ability of *S. asper* and *S. oleraceus* to germinate and thrive may increase their competitiveness, potentially leading to greater weed pressure in crops or natural ecosystems. Consequently, these species could present challenges for management, especially in saline-prone areas.

Overall, our findings provide valuable evidence of the salinity tolerance of *S. asper* and *S. oleraceus*, highlighting their potential to germinate and persist under high salt stress. This understanding can inform strategic weed management approaches, taking into account the unique adaptability of these species. Further research should explore the physiological and phenotypic responses of both species to salinity, as well as management strategies in environments with varying salt concentrations.

### 3.4. Effect of Burial Depth on Seedling Emergence

**Figure 5:**
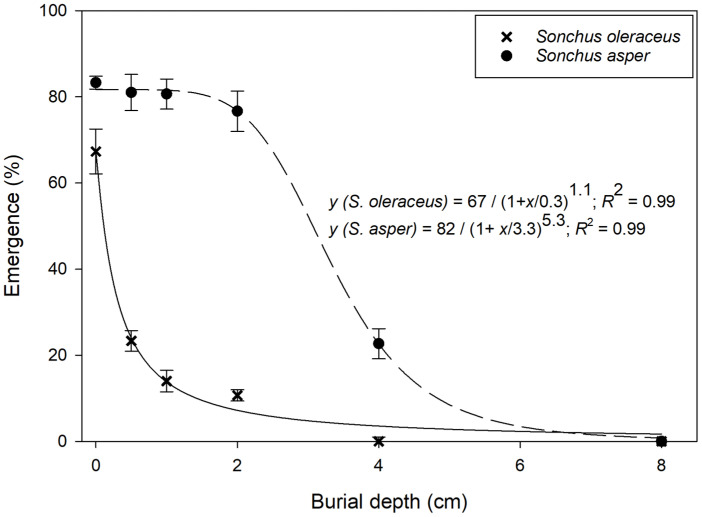
Effect of seed burial depth on the emergence of *Sonchus oleraceus* and *Sonchus asper*. Error bars represent the standard error of mean (*n* = 6). Data were subjected to a three-parametric logistic curve.

Seedling emergence data for *Sonchus* spp. were fitted to a three-parametric logistic model. On the soil surface, seed germination was greater for *S. asper* compared to *S. oleraceus* (Figure 5). The model predicted that the maximum emergence of *S. oleraceus* and *S. asper* was 67% and 82%, respectively. The burial depth required to inhibit 50% emergence of *S. oleraceus* and *S. asper* was 0.3 cm and 3.3 cm, respectively. This research demonstrates that *S. oleraceus* exhibited greater emergence from the soil surface than seeds placed at deeper soil layers. Seedling emergence of *S. oleraceus* decreased as planting depth increased, with no emergence from seeds placed at a 4 cm depth. Our results suggest that no-till farming and shallow tillage could enhance the seedling emergence of both species, especially *S. oleraceus*.

Tillage is a widely used cultural practice to manage weed populations by disturbing the soil and influencing the depth at which weed seeds are buried [23]. However, as highlighted by our results and supported by other studies, not all weed species respond similarly to tillage [24]. For *S. asper*, the ability to germinate from depths up to 5 cm means that shallow tillage may promote its emergence, as seeds near the surface or within the top few centimeters of soil remain viable and capable of germinating. In contrast, *S. oleraceus*’s limited ability to germinate from deeper soil layers makes it more susceptible to burial at greater depths, a factor that could be exploited in deep tillage systems.

These observations suggest that no-till or shallow tillage systems may inadvertently favor *S. asper* by leaving seeds within their preferred germination range. Consequently, strategic deep tillage could be a more effective method for controlling this species, as it would bury seeds beyond their viable emergence depth. Conversely, shallow tillage might be more beneficial for controlling *S. oleraceus*, as this species struggles to emerge from even moderate depths.

The effectiveness of tillage also depends on other factors, such as water availability and competition for growing space. Previous research emphasized that while a large seed bank in appropriate soil depths can enhance weed establishment, factors like moisture and space are equally critical for population expansion [19]. This is particularly relevant for *S. asper*, which has demonstrated greater drought tolerance when seeds are closer to the surface [4]. In drier conditions, shallow-buried *S. asper* may gain a competitive advantage over other species, further complicating weed management efforts in minimum tillage systems.

Given *S. asper*’s greater adaptability to different soil depths, darkness, and stress conditions, an integrated management strategy combining strategic tillage and competitive tactics, such as crop rotation and cover cropping, and timely herbicide application may prove more effective. For instance, rotating tillage practices with other weed control methods, like herbicide application and crop competition, could help prevent *S. asper* from establishing dominance in no-till systems.

Overall, this burial study highlights the need for tailored tillage strategies based on the biological characteristics of specific weed species. For *S. asper*, shallow tillage or no-till systems may increase the risk of weed emergence, suggesting that effective control is required in no-till systems. In contrast, *S. oleraceus* is more susceptible to burial, making shallow tillage a viable strategy for its management. Understanding these dynamics allows for more effective weed control practices that minimize weed emergence while supporting crop yields.

This study demonstrates the germination behavior of *S. oleraceus* and *S. asper* in response to various environmental conditions. The findings highlight the significance of temperature, light, osmotic stress, salt stress, and burial depth for developing integrated management solutions for both invasive weeds, as supported by other researchers for the management of different weeds [25,26,27,28,29,30]. Results demonstrated that *S. oleraceus* is more sensitive to lower temperatures than *S. asper*, suggesting *S. olerceus*’s establishment could be limited in colder regions. *Sonchus oleraceus* showed reduced germination under dark conditions. Therefore, practices such as strategic tillage and mulching can reduce its emergence. The higher drought resilience in *S. asper* suggests it may dominate in water-limited environments, exacerbating its invasiveness. The response of both species to salt stress may increase its invasiveness in saline soils. Burial depth study suggests that *S. asper* can emerge from greater depths than *S. oleraceus*, which provides an opportunity to control this weed through strategic tillage. Future research should investigate the physiological mechanisms for the varying behavior of these weeds in relation to different environmental conditions. Understanding these mechanisms could lead to innovative management solutions tailored to specific agricultural landscapes of eastern Australia.

## Data Availability

All relevant data are within the manuscript.

## References

[1] McCarren K.L., Scott J.K. (2013). Host range and potential distribution of *Aceria thalgi* (Acari: Eriophyidae): A biological control agent for *Sonchus* species. Aust. J. Entomol..

[2] Hutchinson I., Colosi J., Lewin R.A. (1984). The biology of Canadian weeds: 63. *Sonchus asper* (L.) Hill and *S. oleraceus* L. Can. J. Plant Sci..

[3] Steinmaus S.J., Prather T.S., Holt J.S. (2000). Estimation of base temperatures for nine weed species. J. Exp. Bot..

[4] Cordeau S., Wayman S., Reibel C., Strbik F., Chauvel B., Guillemin J.P. (2018). Effects of drought on weed emergence and growth vary with the seed burial depth and presence of a cover crop. Weed Biol. Manag..

[5] Manalil S., Ali H.H., Chauhan B.S. (2020). Interference of annual sowthistle (*Sonchus oleraceus*) in wheat. Weed Sci..

[6] Guerra J.G., Cabello F., Fernández-Quintanilla C., Peña J.M., Dorado J. (2022). Plant functional diversity is affected by weed management through processes of trait convergence and divergence. Front. Plant Sci..

[7] Chauhan B.S., Jha P. (2020). Glyphosate resistance in *Sonchus oleraceus* and alternative herbicide options for its control in southeast Australia. Sustainability.

[8] Mobli A., Sahil, Yadav R., Chauhan B.S. (2020). Enhanced weed-crop competition effects on growth and seed production of herbicide-resistant and herbicide-susceptible annual sowthistle (*Sonchus oleraceus*). Weed Biol. Manag..

[9] Rashid A., Newman J.C., O’Donovan J.T., Robinson D., Maurice D., Poisson D., Hall L.M. (2003). Sulfonylurea herbicide resistance in *Sonchus asper* biotypes in Alberta, Canada. Weed Res..

[10] Broster J.C., Chambers A.J., Weston L.A., Walsh M.J. (2022). Annual ryegrass (*Lolium rigidum*), wild oats (*Avena* spp.) and sowthistle (*Sonchus oleraceus*) are the most commonly occurring weeds in New South Wales cropping fields. Agronomy.

[11] Mahajan G., Chauhan B.S. (2020). Weed biology—A required foundation for effective weed management. Weeds J. Asian-Pac. Weed Sci. Soc..

[12] Singh A., Mahajan G., Chauhan B.S. (2022). Germination ecology of wild mustard (*Sinapis arvensis* L.) and its implications for weed management. Weed Sci..

[13] Manalil S., Ali H.H., Chauhan B.S. (2018). Germination ecology of *Sonchus oleraceus* L. in the northern region of Australia. Crop Past. Sci..

[14] Collier D.E., Cummins W.R. (1990). The effects of low growth and measurement temperature on the respiratory properties of five temperate species. Ann. Bot..

[15] Gresta F., Cristaudo A., Onofri A., Restuccia A., Avola G. (2010). Germination response of four pasture species to temperature, light, and post-harvest period. Plant Biosyst..

[16] Jacobs T.M., Tubeileh A.M., Steinmaus S.J. (2024). Thermal-time hazard models of seven weed species germinability following heat treatment. Agronomy.

[17] Khan Z., Albrecht M., Traveset A. (2013). Salt application as an effective measure to control ruderal invaders threatening endangered halophytic plant species app. Veg. Sci..

[18] Neckár K., Brant V., Necasová M., Nováková K., Venclová V. (2008). Germination of weed species from Asteraceae family under water deficit conditions. J. Plant Dis. Prot..

[19] Chauhan B.S., Gill G., Preston C. (2006). Factors affecting seed germination of annual sowthistle (*Sonchus oleraceus*) in southern Australia. Weed Sci..

[20] Widderick M.J., Walker S.R., Sindel B.M., Bell K.L. (2010). Germination, emergence, and persistence of *Sonchus oleraceus*, a major crop weed in subtropical Australia. Weed Biol. Manag..

[21] Gkotzamani A., Ipsilantis I., Menexes G., Katsiotis A., Mattas K., Koukounaras A. (2024). The impact of salinity in the irrigation of a wild underutilized leafy vegetable, *Sonchus oleraceus* L. Plants.

[22] Mawalagedera S., Gould K.S. (2015). Chilling and salinity increase extractable antioxidants in cell suspension cultures of the sow thistle, *Sonchus oleraceus* L. Plant Cell Tissue Organ Cult..

[23] de La Fuente E.B., Suárez S.A., Ghersa C.M., León R.J.C. (1999). Soybean weed communities: Relationships with cultural history and crop yield. Agron. J..

[24] Chauhan B.S., Gill G., Preston C. (2006). Seedling recruitment pattern and depth of recruitment of 10 weed species in minimum tillage and no-till seeding systems. Weed Sci..

[25] Pegtel D.M. (1972). Effects of temperature and moisture on the germination of two ecotypes of *Sonchus arvensis* L. Acta Bot. Neerl..

[26] Vanijajiva O. (2014). Effect of ecological factors on seed germination of alien weed *Tridax procumbens* (Asteraceae). J. Agric. Ecol. Res..

[27] Tang W., Guo H., Yin J., Ding X., Xu X., Wang T., Yang C., Xiong W., Zhong S., Tao Q. (2022). Germination ecology of *Chenopodium album* L. and implications for weed management. PLoS ONE.

[28] Bittencourt H.V.H., Bonome L.T.S., Trezzi M.M., Vidal R.A., Lana M.A. (2017). Seed germination ecology of *Eragrostis plana*, an invasive weed of South American pasture lands. S. Afr. J. Bot..

[29] Shoab M., Tanveer A., Khaliq A., Ali H.H. (2012). Effect of seed size and ecological factors on germination of *Emex spinosa*. Pak. J. Weed Sci. Res..

[30] Scherner A., Melander B., Jensen P.K., Kudsk P., Avila L.A. (2017). Germination of winter annual grass weeds under a range of temperatures and water potentials. Weed Sci..

